# The clinical epidemiology of fatigue in newly diagnosed heart failure

**DOI:** 10.1186/s12872-017-0555-9

**Published:** 2017-05-11

**Authors:** Brent A. Williams

**Affiliations:** Geisinger Health System, 100 N. Academy Avenue, Danville, PA 17822 USA

**Keywords:** Heart failure, Symptoms, Fatigue, Prognosis, Quality of life

## Abstract

**Background:**

Fatigue is a common and distressing but poorly understood symptom among patients with heart failure (HF). This study sought to evaluate the prevalence, predictors, and prognostic value of clinically documented fatigue in newly diagnosed HF patients from the community.

**Methods:**

This retrospective cohort study consisted of 12,285 newly diagnosed HF patients receiving health care services through the Geisinger Health System, with passive data collection through electronic medical records (EMR). Incident HF, fatigue, and other study variables were derived from coded data within EMRs. A collection of 87 candidate predictors were evaluated to ascertain the strongest independent predictors of fatigue using logistic regression. Patients were followed for all-cause mortality for an average of 4.8 years. The associations between fatigue and 6-month, 12-month, and overall mortality were evaluated via Cox proportional hazards regression models.

**Results:**

Clinically documented fatigue was found in 4827 (39%) newly diagnosed HF patients. Depression demonstrated the strongest association with fatigue. Fatigue was often part of a symptom cluster, as other HF symptoms including dyspnea, chest pain, edema, syncope, and palpitations were significant predictors of fatigue. Volume depletion, lower body mass index, and abnormal weight loss were also strong predictors of fatigue. Fatigue was not significantly associated with either 6-month (HR = 1.12, *p* = 0.16) or overall mortality (HR = 1.00, *p* = 0.89) in adjusted models.

**Conclusions:**

Fatigue is a commonly documented symptom among newly diagnosed HF patients, and its origins may lie in both psychologic and physiologic factors. Though fatigue did provide a prognostic signal in the short-term, this was largely explained by physiologic confounders. Proper therapeutic remediation of fatigue in HF relies on identifying underlying factors.

## Background

Heart failure (HF) is often a highly symptomatic condition, with shortness of breath, fatigue, chest pain, edema, and syncope being the most commonly reported symptoms [1–7]. These symptoms impair quality of life, drive the need for HF-related hospitalizations, inhibit individuals’ ability to function physically and perform activities of daily living, and in many cases signal a poor prognosis in the HF condition [1, 8]. Fatigue in particular has often been cited as the most common and distressing symptom associated with HF [3, 5–7, 9]. However, as instruments for measuring the quantitative and qualitative aspects of fatigue have not been studied extensively in HF nor reached widespread incorporation in clinical practice, the true burden imposed by fatigue in HF has been challenging to assess [5]. Furthermore, the origins of fatigue in HF are not well understood and several contributing etiologies are possible which confounds proper application of therapy [3–7, 9–12]. Previous studies have had limited ability to evaluate these several potential etiologic factors collectively. Lastly, whether fatigue signals a hastened mortality independent of other clinical factors remains unresolved.

Accordingly, the current study sought to expand knowledge on the clinical epidemiology of fatigue in a community-based cohort of newly diagnosed HF patients receiving primary care and other health care services through a single health care system. In particular, this study employed the extensive electronic medical record (EMR) data repository of the study institution to (1) estimate the prevalence of clinically documented fatigue in a large cohort of patients with newly diagnosed HF; (2) identify independent predictors of fatigue with a goal of better understanding possible etiologies; and (3) determine whether fatigue is independently associated with post-diagnosis mortality and whether the prognostic effect varies by time since diagnosis.

## Methods

### Geisinger Health System

This study consists of 12,285 individuals with newly diagnosed HF receiving primary care and other health care services through the Geisinger Health System (Geisinger) between January 1, 2001, and December 31, 2013. The parent study was approved by the Geisinger Institutional Review Board who allowed a waiver of patient consent due to the retrospective nature of the study. Geisinger consists of numerous ambulatory clinics providing both primary and specialty care, several community hospitals, and a full-service insurance company. Since 2001, Geisinger has provided health care services to over three million people throughout central and northeast Pennsylvania. In recent years, Geisinger physicians and advanced practitioners have served approximately 500,000 patients annually through its network of clinics and hospitals. All outpatient and inpatient facilities are linked through the EMR system, EpicCare. The Geisinger EMR data repository contains information on all 3+ million patients including detailed demographics, vital signs, social history (e.g. smoking, alcohol), diagnoses, procedures, problem lists, medical history, medications, laboratory results, and billing information from nearly all outpatient and inpatient encounters at Geisinger since 2001. Importantly, the aforementioned data elements are contained within fixed fields with a standardized structure and content across all Geisinger facilities, facilitating data extraction and organization for large research data sets as described here.

### Heart failure patient population

All patients meeting the following criteria were included in this study: (1) receiving health care services through Geisinger for at least a 2-year period between January 2001 and December 2013; (2) being free of HF for at least 2 years following the first Geisinger encounter as judged by the absence of HF diagnosis codes at all encounters during this interval; (3) having at least one primary care encounter at Geisinger during the HF-free period; and (4) developing incident HF as defined by the presence of HF diagnosis codes at either one inpatient or two outpatient encounters after the HF-free period. These inclusion criteria are designed to capture a representative, community-based sample of HF patients receiving all or most of their health care through Geisinger. For this study, *primary care* encounters are defined as clinical visits to a department of family medicine or general internal medicine within Geisinger. The 2-year HF-free period is considered an optimum blanking period for valid exclusion of pre-existing HF among individuals entering the Geisinger EMR system. HF diagnoses are based on the following International Classification of Diseases – Ninth Revision (ICD-9) codes: 398.91, 402.X, 404.X, or 428.X. ICD-9 codes can be found within multiple EMR domains, including encounter diagnoses (primary or secondary), problems lists, and billing. The date of HF diagnosis was defined as the first day an HF diagnosis code was found.

### Other study variables

Fatigue status (yes/no) as of the date of diagnosis was determined by the presence of any of the following ICD-9 codes at any encounter either prior to, or up to 90 days following the diagnosis date: 780.7; 780.71; 780.79. Fatigue was documented in the EMR as a discrete diagnosis either by clinical staff directly interacting with the patient, or by billing coding specialists during post-encounter review of clinical notes. Other medical history variables were defined in an analogous manner according to appropriate ICD-9 codes. Age and smoking status were determined as of the diagnosis date. Vital signs and laboratory results included were those measured closest, but prior to, the diagnosis date. Medications include those with an active prescription on the diagnosis date or within 90 days following. All-cause mortality was derived by cross-referencing the master list of Geisinger patients with files from the Social Security Administration. Deceased patients are flagged and date of death recorded.

### Data analysis strategy

Baseline characteristics at the diagnosis date, including demographics, physical examination findings, medical history, laboratory results, and medications are reported across fatigue status as percentages for categorical variables and medians with interquartile ranges for continuous variables. Differences in categorical variables were tested for statistical significance by chi-square tests, and differences in continuous variables by Wilcoxon rank sum tests as many of the continuous variables were not normally distributed.

Independent predictors of fatigue were determined by multivariable logistic regression with fatigue status as the binary dependent variable. A forward stepwise variable selection algorithm was employed to identify those variables with the strongest independent associations with fatigue. The 87 candidate variables considered for inclusion in the final model are those listed in Tables 1, 2 and 3. The collection of candidate variables was chosen based on a combination of potential relevance in explaining fatigue, typical comorbid conditions which accompany HF, and data availability. For this analysis, laboratory values were divided into multi-level nominal categories to ease interpretation while accounting for possible non-linear associations with fatigue status. Separate categories were created for missing lab values. Multivariable models developed from a large data set such as considered here tend to find an abundance of predictors statistically significant at typical *p*-value thresholds despite having relatively small effect sizes and thus questionable clinical relevance. To circumvent this potential difficulty, a more stringent *p*-value threshold of 0.001 for model inclusion was employed. Furthermore, a model term for the number of days between the first EMR-documented Geisinger encounter and the HF diagnosis date (encounter time) was forced into the final model as a control term. Encounter time introduces a potential information bias into the study design as a positive correlation exists between encounter time and many diagnoses. This information bias is partly controlled by including encounter time in the final model. Upon achieving a final model, significant predictors are reported according to the rank order of their associated Wald chi-square test statistic (from highest to lowest) which is qualitatively equivalent but more quantitatively informative than ranking according to *p*-value magnitude (from lowest to highest). The discriminatory ability of the final model was evaluated by the c-statistic.

**Table 1:** Baseline characteristics of newly diagnosed heart failure patients stratified by fatigue status at baseline

	All patients (*n* = 12,285)	No Fatigue (*n* = 7458)	Fatigue (*n* = 4827)	*p*-value
Demographics				
Age, years	76 (66, 83)	75 (65, 82)	77 (67, 84)	<0.001
Female, %	51	47	57	<0.001
Smoking history, %	56	56	54	0.030
Physical examination				
Body mass index, kg/m^2^	30 (26, 35)	30 (26, 36)	29 (25, 35)	<0.001
Systolic BP, mm Hg	128 (117, 142)	130 (118, 144)	128 (116, 142)	0.005
Diastolic BP, mm Hg	70 (62, 80)	70 (62, 80)	70 (60, 78)	<0.001
Heart rate, bpm	75 (66, 84)	74 (64, 84)	76 (66, 84)	0.042
Medical history				
Abnormal weight loss, %	11	8	15	<0.001
Anemia, %	48	41	59	<0.001
Anxiety disorder, %	11	9	15	<0.001
Aortic aneurysm, %	7	6	8	<0.001
Atrial fibrillation, %	37	35	40	<0.001
Cancer, %	30	27	33	<0.001
Cardiomyopathy, %	14	14	14	0.272
Cerebrovascular disease, %	34	29	42	<0.001
Chest pain, %	53	47	63	<0.001
Chronic lung disease, %	39	36	43	<0.001
Conduction disorder, %	16	14	19	<0.001
Coronary atherosclerosis, %	60	59	61	0.012
Dementia, %	6	5	8	<0.001
Depression, %	26	19	36	<0.001
Diabetes, %	44	44	44	0.616
Dyspnea, %	62	55	71	<0.001
Edema, %	43	39	50	<0.001
Gastroesophageal reflux, %	43	37	53	<0.001
Gout, %	12	12	13	0.006
Hypercholesterolemia, %	76	74	80	<0.001
Hyperpotassemia, %	8	7	11	<0.001
Hypertension, %	88	86	90	<0.001
Hyperthyroidism, %	3	2	3	0.003
Hypopotassemia, %	17	13	22	<0.001
Hypothyroidism, %	26	22	31	<0.001
Kidney disease, %	31	28	36	<0.001
Liver disease, %	4	3	5	<0.001
Myocardial infarction, %	25	24	26	<0.001
Palpitations, %	11	9	15	<0.001
Peripheral vascular disease, %	27	23	33	<0.001
Pulmonary embolism, %	5	5	7	<0.001
Pulmonary hypertension, %	14	13	16	<0.001
Sleep apnea, %	15	13	17	<0.001
Syncope, %	31	23	43	<0.001
Tachycardia, %	9	8	12	<0.001
Valve disease, %	39	36	43	<0.001
Venous thromboembolism, %	7	6	9	<0.001
Vitamin D deficiency, %	12	8	18	<0.001
Volume depletion, %	11	7	17	<0.001

*BP* Blood pressure

**Table 2:** Laboratory values of newly diagnosed heart failure patients stratified by fatigue status at baseline

	All patients (*n* = 12,285)	No Fatigue (*n* = 7458)	Fatigue (*n* = 4827)	*p*-value
Alanine aminotransferase, IU/L	20 (15, 30)	21 (15, 30)	20 (14, 29)	<0.001
Albumin, g/dl	4.0 (3.6, 4.2)	4.0 (3.7, 4.2)	3.9 (3.6, 4.2)	<0.001
Alkaline phosphatase, U/L	78 (63, 99)	78 (64, 98)	77 (63, 99)	0.43
Aspartate aminotransferase, U/L	25 (20, 32)	25 (20, 32)	25 (20, 32)	0.51
Bicarbonate, mEq/l	27 (25, 30)	27 (25, 30)	27 (25, 30)	<0.01
Bilirubin, mg/dl	0.5 (0.3, 0.7)	0.5 (0.4, 0.7)	0.5 (0.3, 0.7)	<0.001
Blood urea nitrogen, mg/dl	21 (16, 28)	21 (16, 28)	21 (16, 28)	0.32
Calcium, mg/dl	9.2 (8.9, 9.6)	9.2 (8.9, 9.6)	9.2 (8.8, 9.5)	<0.001
Chloride, mmol/l	102 (99, 105)	102 (99, 105)	102 (99, 105)	0.04
Creatinine, mg/dl	1.0 (0.8, 1.3)	1.0 (0.8, 1.3)	1.0 (0.8, 1.3)	0.64
Glucose, mg/dl	110 (95, 141)	111 (95, 142)	110 (95, 139)	0.23
HDL cholesterol, mg/dl	47 (38, 57)	46 (38, 56)	47 (38, 58)	<0.001
Hematocrit, %	38.2 (34.3, 41.8)	38.8 (34.8, 42.3)	37.5 (33.6, 41.1)	<0.001
Hemoglobin, g/dl	12.8 (11.4, 14.1)	13.0 (11.6, 14.3)	12.6 (11.2, 13.8)	<0.001
LDL cholesterol, mg/dl	87 (66, 111)	87 (67, 110)	86 (66, 111)	0.17
MCH, pg	30.5 (29.2, 31.8)	30.5 (29.2, 31.8)	30.5 (29.1, 31.8)	0.63
MCHC, g/dl	33.7 (32.9, 34.3)	33.7 (32.9, 34.3)	33.6 (32.8, 34.3)	<0.001
Mean corpuscular volume, mcm^3^	90.7 (87.3, 94.2)	90.6 (87.2, 94.0)	90.7 (87.3, 94.4)	0.07
Mean platelet volume, fl	9.4 (8.2, 10.6)	9.4 (8.3, 10.6)	9.4 (8.1, 10.5)	<0.01
Neutrophil:lymphocyte	3.6 (2.3, 6.2)	3.6 (2.3, 6.0)	3.6 (2.4, 6.3)	0.19
Platelet count, ×10^3^/mcl	223 (178, 279)	224 (179, 278)	222 (178, 280)	0.18
Potassium, mEq/l	4.2 (3.9, 4.6)	4.3 (3.9, 4.6)	4.2 (3.9, 4.6)	<0.001
Protein, g/dl	6.9 (6.4, 7.2)	6.9 (6.5, 7.3)	6.8 (6.4, 7.2)	<0.001
RDW, %	14.2 (13.4, 15.3)	14.1 (13.4, 15.2)	14.3 (13.5, 15.5)	<0.001
Red blood cell count, ×10^6^/mcl	4.24 (3.77, 4.65)	4.30 (3.85, 4.70)	4.15 (3.68, 4.56)	<0.001
Sodium, mmol/l	139 (137, 141)	139 (137, 141)	139 (137, 141)	<0.001
Total cholesterol, mg/dl	165 (139, 194)	165 (139, 194)	165 (138, 195)	0.34
Triglycerides, mg/dl	125 (89, 178)	125 (89, 180)	123 (89, 175)	0.04
White blood cell, ×10^3^/mcl	7.82 (6.36, 9.87)	7.85 (6.40, 9.84)	7.79 (6.27, 9.94)	0.43

*HDL* High density lipoprotein, *LDL* Low density lipoprotein, *MCH* Mean corpuscular hemoglobin, *MCHC* Mean corpuscular hemoglobin concentration, *RDW* Red cell distribution width

**Table 3:** Medications of newly diagnosed heart failure patients stratified by fatigue status at baseline

	All patients (*n* = 12,285)	No Fatigue (*n* = 7458)	Fatigue (*n* = 4827)	*p*-value
ACE inhibitor, %	68	67	68	0.34
Angiotensin receptor blocker, %	21	19	24	<0.001
Antiarrhythmic type III, %	19	18	21	<0.001
Anticoagulants, %	38	36	40	<0.001
Antiplatelet, %	27	25	29	<0.001
Aspirin, %	76	74	79	<0.001
Beta blocker, %	80	78	82	<0.001
Calcium channel blocker, %	45	43	49	<0.001
Digoxin, %	19	19	19	0.61
Diuretic, %	88	86	90	<0.001
Nitrates, %	47	45	50	<0.001
Statin, %	66	65	68	<0.001
Antidepressant, %	41	34	54	<0.001

*ACE* Angiotensin Converting Enzyme

For evaluating the prognostic impact of fatigue, a time-to-event variable was created as the number of days from the HF diagnosis date until death or December 31, 2013, the last date survival status was known with confidence. Patients not known to have died were censored on this date. Kaplan-Meier survival estimates were calculated at various time points following diagnosis both overall and stratified by fatigue status. Differences in survival by fatigue status were evaluated for statistical significance by the log rank test. Visual inspection of crude survival curves suggested an early divergence within the first six to 12 months following diagnosis which did not appreciably worsen beyond 12 months, suggesting fatigue may portend a poor short-term prognosis but lose its predictive ability long-term. Accordingly, two additional survival endpoints were considered: death within 6 months of diagnosis and death within 12 months of diagnosis. Cox proportional hazards models were developed to evaluate the association between fatigue and mortality endpoints. Hazard ratios and 95% confidence intervals are reported for all models. An adjusted Cox model included all independent predictors of fatigue as identified by the aforementioned logistic regression model. In survival models, interactions between fatigue-gender and fatigue-depression were evaluated given the strong associations between these variables.

## Results

### Characteristics of incident HF at diagnosis

Among 12,285 newly diagnosed HF cases, 4195 (34%) were inpatient diagnoses and 8090 (66%) were outpatient diagnoses. The average (SD) number of years between the first Geisinger encounter and diagnosis date was 6.6 (3.1) years. The median age of new HF cases was 76 years and 51% were female. HF cases had large amounts of cardiovascular comorbidity with 60% having coronary atherosclerosis, 25% prior myocardial infarction, 34% cerebrovascular disease, and 37% atrial fibrillation. Clinically documented symptoms near, or prior to, the HF diagnosis date were highly prevalent with 62% having documented dyspnea, 53% chest pain, 43% edema, and 31% syncope.

Fatigue was clinically documented in 4827 (39%) of the incident HF cases. Patients with fatigue were on average older, more likely to be female, had more cardiovascular and non-cardiovascular comorbidity, and more additional symptoms including dyspnea, chest pain, edema, syncope, and palpitations (Table 1). Though absolute differences were small, patients with fatigue had several worse laboratory parameters, including markers of nutrition (albumin, protein) and multiple red blood cell indices (hemoglobin, hematocrit, red blood cell count) (Table 2). Patients with fatigue were also more likely to be prescribed several medications, though absolute differences across fatigue status groups were small (Table 3).

### Predictors of fatigue

Out of 87 candidate predictors, 18 were independently associated with fatigue at a multivariable *p*-value threshold of 0.001 (Table 4). Significant predictors of fatigue are listed in Table 4 from strongest to weakest according to the Wald chi-square statistic from the final multivariable model. Depression had the strongest association with fatigue, being associated with an 80% increased likelihood of fatigue. Other symptoms were also strongly associated with fatigue including syncope (61% increased likelihood of fatigue), dyspnea (33%), chest pain (31%), palpitations (32%), and edema (19%). Several possible indicators of volume regulation, cachexia, and/or frailty were also strongly associated with fatigue including volume depletion (56%), abnormal weight loss (39%), and body mass index (7% higher likelihood of fatigue per 5 kg/m^2^ lower BMI). Female gender (37%) and anemia (32%) were also among the strongest predictors of fatigue. The c-statistic for the full model was 0.734 (95% CI: 0.725, 0.743).

**Table 4:** Multivariable predictors of fatigue among newly diagnosed heart failure patients

	Odds ratio^a^ (95% CI)	Wald chi-square
1) Depression	1.80 (1.64, 1.97)	152.9
2) Syncope	1.61 (1.47, 1.76)	110.4
3) Female	1.37 (1.26, 1.50)	50.6
4) Volume depletion	1.56 (1.37, 1.78)	44.3
5) Dyspnea	1.33 (1.22, 1.46)	39.8
6) Gastroesophageal reflux	1.30 (1.20, 1.42)	38.7
7) Chest pain	1.31 (1.20, 1.43)	37.6
8) Anemia	1.32 (1.21, 1.45)	37.2
9) BMI (per 5 kg/m^2^ increase)	0.93 (0.90, 0.96)	25.0
10) Abnormal weight loss	1.39 (1.21, 1.59)	23.0
11) Sleep apnea	1.35 (1.19, 1.53)	23.0
12) Palpitations	1.32 (1.16, 1.49)	18.2
13) Cerebrovascular disease	1.20 (1.10, 1.31)	17.0
14) Vitamin D deficiency	1.30 (1.15, 1.48)	16.8
15) Edema	1.19 (1.09, 1.30)	16.2
16) Hematocrit (H-NL v. NL)	1.34 (1.06, 1.70)	15.6
17) Hypopotassemia	1.21 (1.09, 1.35)	11.9
18) Age (per 5 year increase)	1.03 (1.02, 1.05)	11.1

*H-NL* High-Normal, *NL* Normal, *BMI* Body Mass Index

^a^Adjusted for number of days from first encounter to diagnosis date

### Prognostic value of fatigue

**Fig. 1:**
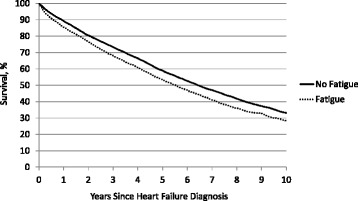
Post-diagnosis survival by fatigue status among newly diagnosed heart failure patients

Among the 12,285 incident HF cases, 5679 (46%) were known to have died by December 31, 2013. Average follow-up time among known survivors was 4.8 (±3.1) years. Kaplan-Meier survival estimates at 6 months, 1 year, 5 years, and 10 years following diagnosis were 93%, 88, 57 and 31%, respectively. Survival curves stratified by fatigue status at time of diagnosis showed early divergence within the first year following diagnosis with little additional divergence over subsequent follow-up (Fig. 1). Unadjusted hazard ratios for fatigue at diagnosis were 1.49 (1.31, 1.70; *p* < 0.001), 1.39 (1.26, 1.54; *p* < 0.001), and 1.20 (1.14, 1.27; *p* < 0.001) for 6-month, 12-month, and overall mortality, respectively. After adjustment for the predictors of fatigue identified earlier, hazard ratios were greatly attenuated: 1.12 (0.96, 1.30; *p* = 0.16) for 6-month mortality; 1.07 (0.95, 1.21; *p* = 0.26) for 12-month mortality; and 1.00 (0.94, 1.06; *p* = 0.89) for overall mortality (Table 5). Much of the attenuation in adjusted models could be attributed to anemia, dyspnea, lower body mass index, volume depletion, and abnormal weight loss, as these variables were strongly associated with both fatigue and short-term mortality (Tables 4 & 5). No significant interactions were observed between fatigue-gender and fatigue-depression on any survival endpoints (all *p* > 0.4).

**Table 5:** Multivariable Cox proportional hazards models for 6-month, 12-month, and overall mortality

	6-Month HR (95% CI)	12-Month HR (95% CI)	Overall HR (95% CI)
Fatigue			
Unadjusted	1.49 (1.31, 1.70)	1.39 (1.26, 1.54)	1.20 (1.14, 1.27)
Adjusted	1.12 (0.96, 1.30)	1.07 (0.95, 1.21	1.00 (0.94, 1.06)
1) Age (per 5 year increase)	1.19 (1.15, 1.25)	1.19 (1.15, 1.23)	1.25 (1.23, 1.26)
2) Anemia	1.60 (1.35, 1.90)	1.46 (1.28, 1.67)	1.18 (1.11, 1.26)
3) Dyspnea	1.62 (1.37, 1.92)	1.48 (1.30, 1.69)	1.18 (1.12, 1.26)
4) BMI (per 5 kg/m^2^ increase)	0.84 (0.79, 0.90)	0.82 (0.77, 0.86)	0.92 (0.89, 0.94)
5) Hypopotassemia	1.41 (1.20, 1.67)	1.32 (1.15, 1.50)	1.17 (1.08, 1.26)
6) Volume depletion	1.43 (1.19, 1.72)	1.35 (1.16, 1.56)	1.28 (1.18, 1.39)
7) Abnormal weight loss	1.40 (1.16, 1.68)	1.41 (1.22, 1.63)	1.34 (1.23, 1.46)
8) Cerebrovascular disease	1.24 (1.07, 1.43)	1.28 (1.14, 1.44)	1.25 (1.18, 1.32)
9) Palpitations	0.73 (0.58, 0.94)	0.71 (0.59, 0.86)	0.72 (0.65, 0.80)
10) Female	0.84 (0.72, 0.98)	0.84 (0.75, 0.95)	0.81 (0.77, 0.86)
11) Syncope	0.86 (0.73, 1.00)	0.87 (0.77, 0.99)	0.93 (0.87, 0.99)
12) Sleep apnea	0.77 (0.59, 1.00)	0.89 (0.73, 1.08)	0.96 (0.87, 1.06)
13) Depression	1.14 (0.97, 1.34)	1.20 (1.05, 1.36)	1.18 (1.10, 1.26)
14) Edema	1.12 (0.97, 1.30)	1.24 (1.10, 1.38)	1.21 (1.14, 1.28)
15) Chest pain	0.92 (0.79, 1.07)	0.97 (0.86, 1.09)	0.88 (0.83, 0.93)
16) Vitamin D deficiency	0.90 (0.74, 1.10)	0.94 (0.81, 1.11)	1.12 (1.01, 1.23)
17) Hematocrit (H-NL vs. NL)	0.79 (0.45, 1.40)	0.93 (0.62, 1.39)	1.04 (0.87, 1.25)
18) Gastroesophageal reflux	0.98 (0.85, 1.14)	0.92 (0.82, 1.04)	0.94 (0.89, 1.00)

*H-NL* High-Normal, *NL* Normal, *BMI* Body Mass Index

## Discussion

This study sought to evaluate the clinical epidemiology of fatigue in a large cohort of newly diagnosed HF patients from a non-clinical trial, community-based setting. The extensive EMR data repository of the Geisinger Health System was utilized to provide a detailed perspective on the prevalence, predictors, and prognostic value of clinically documented fatigue in a large HF population receiving primary care and other health care services through a single health care system. We found that 39% of incident HF patients had clinically documented fatigue at the time of diagnosis. Fatigue was often part of a symptom cluster, as individuals with fatigue were also significantly more likely to have documented dyspnea, chest pain, edema, syncope, and palpitations. The variable showing the strongest association with fatigue was depression, suggesting a strong psychological component to fatigue in HF. Fatigue was also strongly associated with other factors possibly indicative of fluid homeostasis, cachexia, and/or frailty, such as volume depletion, lower body mass index, and abnormal weight loss. Fatigue was not independently associated with either short- or long-term mortality after considering the strongest correlates of fatigue.

Heart failure is often a very symptomatic condition, and the presence and severity of symptoms often contribute to its diagnosis [4]. Fatigue in particular has been noted as a very common symptom in HF, but estimating its true prevalence is challenging as the fatigue construct is intrinsically difficult to define in an objective manner and no fatigue measurement tool exists that has gained widespread popularity in either the clinical or research settings [2, 5–7]. Prior studies have reported that the prevalence of fatigue in HF ranges from 28 to 59% with variation likely attributable to the method of measurement, specific characteristics of the HF population studied, timing of the measurement with respect to disease exacerbations, and other factors [10, 13–17]. Our estimate of fatigue prevalence in a newly diagnosed HF population (39%) falls within the range observed in previous studies. The definition of fatigue employed in the current study involved observing any one of three fatigue-related ICD-9 codes either near, or prior to, the HF diagnosis date. Of note, fatigue had to be either directly coded into the EMR by clinical staff or abstracted from a clinical note reviewed by a billing coding specialist. Given the ubiquity of fatigue, it seems likely that fatigue documented in the clinical environment resulted from the fatigue being *greater than what is typical or expected* with respect to frequency, severity, duration, and/or ease of provocation, but we are unable to validate this supposition. Evaluating prevalence estimates from prior studies in concert with the employed fatigue definition may help clarify the meaning of clinically documented fatigue in our study population. For instance, Ingle et al. reported that 27% of HF patients had some, a lot, or very much fatigue at rest, while 38% reported fatigue limited their daily activity some, a lot, or very much [15]. Perez-Moreno et al. noted that 43% of HF patients reported fatigue at rest or with slight exertion [10]. Ekman et al. found that 50% of HF patients reported fatigue either walking at normal pace on a flat surface, walking slowly on a flat surface or during washing or dressing, or at rest [17]. Finally, Barnes et al. found that 59% of chronic HF patients were moderately to extremely troubled by fatigue [16].

Identifying the origin(s) of fatigue in HF can also be challenging as fatigue may be a consequence of one or more underlying etiologies. In the context of HF, fatigue may be attributable to peripheral sequelae of the cardiac dysfunction itself, psychological morbidity which often accompanies HF, any of several additional comorbidities often coexisting with HF, or aging [2, 4]. Our analysis of a large number of candidate predictors of fatigue serves not only to identify potential etiologic origins of fatigue but also suggests possible therapeutic opportunities for its alleviation. The variable with the strongest association with fatigue in our analysis was depression, suggesting a strong psychological component to fatigue in HF. Depression is common in HF (26% in our study), and depressive symptoms have significant overlap with several HF-related symptoms, including fatigue [3, 5, 18]. Several previous studies have noted an association between depression and fatigue in HF, but the nature of the causal relationship is not clear and may be bidirectional [6, 18, 19]. In the current study, depression was documented prior to fatigue in 59% of patients with both conditions documented, while fatigue was documented first in 36% (5% on same day). Our results also revealed that fatigue was often part of a symptom cluster, as dyspnea, chest pain, edema, syncope, and palpitations were all independently associated with fatigue. Recognition of symptom clusters in HF is increasing, along with their potential to impact adversely both life quality and expectancy [20–23]. Symptom clusters may reflect a common underlying etiology (e.g. volume overload) causing multiple somatic manifestations and/or an increased awareness of additional symptoms by patient or provider once an initial problematic symptom has been identified [20–24].

We also found volume depletion, lower body mass index, and abnormal weight loss to be among the strongest predictors of fatigue. These factors may reflect some combination of volume dysregulation, cachexia, and/or frailty, all of which are not infrequent in the HF population and signal an advanced stage of disease and an adverse prognosis [4]. These traits may be a direct result of catabolic processes in the skeletal musculature believed to be activated in HF, leading to structural and functional alterations which impede oxygen delivery and utilization in the working muscle, causing muscle weakness, atrophy, and wasting [7, 9, 25, 26]. These aberrations have been hypothesized as a direct cause of fatigue and other symptoms in HF, and importantly, both the muscle abnormalities and fatigue have been shown to improve with exercise training [4, 9, 25–27]. Beyond depression and markers of volume regulation and frailty, other notable independent predictors of fatigue were female gender, anemia, sleep apnea, vitamin D deficiency, and higher age – female gender and anemia in particular have shown to be predictors of fatigue in prior studies [4, 6, 17, 28, 29].

The logic behind evaluating the independent prognostic effect of fatigue (or any symptom) lies in its potential as a signal of some prognostically relevant pathophysiologic process not captured by usual clinical measures. In this regard, our results are largely consistent with prior studies showing that fatigue has at most a small, but perhaps no, independent association with all-cause mortality after adjusting for the strongest correlates of fatigue [10, 11, 15, 17, 29, 30]. Though our results showed fatigue was associated with a 49% increased risk of short-term death within 6 months following HF diagnosis, the effect diminished greatly (HR = 1.12, *p* = 0.16) after controlling for the strongest correlates of fatigue. Notably, this small effect diminished further when evaluating 12-month (HR = 1.07, *p* = 0.26) and overall (HR = 1.00, *p* = 0.97) mortality, suggesting that any prognostic signal provided by fatigue wanes over time. Importantly, our results suggest that the presence of dyspnea (often a marker of congestion), anemia, volume depletion, and a lower body mass index explain much of the increased risk associated with fatigue in unadjusted models. Multiple prior studies have observed no to modest increased mortality risk with various measures of fatigue. In a recent secondary analysis of the CORONA trial (Controlled Rosuvastatin Multinational Trial in Heart Failure), Perez-Moreno et al. showed that the highest level of fatigue was associated with a non-significant 17% increased risk of all-cause mortality (*p* = 0.26) in patients with systolic HF of ischemic origin, while Ekman et al. reported no independent prognostic effect of fatigue among systolic HF patients enrolled in COMET (Carvedilol or Metoprolol European Trial) [10, 17]. Notably, these two studies did show that fatigue was associated with an increased risk of HF hospitalization and worsening HF, respectively [10, 17]. Our work confirms and extends the mortality findings to a community-based cohort with HF of various origins and ejection fractions.

Study limitations derive largely from the retrospective nature of this study originating from EMRs. Study data is derived from usual clinical practice, thus there was no standardized assessment of fatigue or other study variables and variability in inter-coder practices is inevitable. The patient had to either spontaneously report fatigue as a concern during a clinical encounter or clinical staff had to specifically query about fatigue in the absence of any stimulus. Furthermore, clinical staff had to either explicitly document fatigue with an appropriate ICD-9 code or within a clinical note with subsequent abstraction by a coding specialist. These factors may have led to an underestimation of fatigue prevalence. We also had no information on fatigue severity, frequency, duration, or ease of provocation, and thus were unable to evaluate whether predictors or the prognostic relevance of these fatigue attributes differ from what was observed for our binary definition of fatigue. Furthermore, data elements necessarily were restricted to diagnostic codes and other fixed-field elements which restricted data availability. For instance, New York Heart Association class is a popular metric for assessing the symptomatic impact of HF and likely overlaps with fatigue, but was not available in the current study. Nonetheless, this study was able to include a large, real-world community-based cohort with newly diagnosed HF and evaluate a large number of potential correlates of fatigue well in excess of prior studies.

## Conclusion

Fatigue was a commonly documented symptom among individuals with newly diagnosed HF, but less so than other HF symptoms such as dyspnea, chest pain, and edema. Though fatigue does not appear to signal an adverse prognosis independent of other clinically documented attributes in HF, the symptom is common and capable of imposing a significant adverse impact on life enjoyment. Thus, fatigue may be a meritorious therapeutic target in itself. Indeed, HF guidelines and performance measures note regular symptom assessment and management as important components of HF care, but to our knowledge the efficacy of established HF therapies has not been evaluated with respect to fatigue in particular, perhaps related in part to the difficulty in its measurement [4, 31]. Therapeutic remediation of fatigue requires correctly identifying its etiologic origins, and our results suggest depression, volume regulation, cachexia/frailty, and anemia are the strongest potentially modifiable contributors to fatigue in HF. A standardized, extensively validated instrument for measuring the qualitative and quantitative aspects of fatigue may be valuable in both clinical and research settings, especially in the evaluation of new HF therapies.

## Abbreviations

EMR: Electronic medical record
HF: Heart failure
HR: Hazard ratio
ICD-9: International classification of diseases – 9th revision
